# General Practice Patients’ Experiences and Perceptions of the WiserAD Structured Web-Based Support Tool for Antidepressant Deprescribing: Protocol for a Mixed Methods Case Study With Realist Evaluation

**DOI:** 10.2196/42526

**Published:** 2022-12-29

**Authors:** Amy Coe, Jane Gunn, Catherine Kaylor-Hughes

**Affiliations:** 1 Department of General Practice University of Melbourne Melbourne Australia

**Keywords:** antidepressants, primary care, depression, deprescribing, realist evaluation, online support tool, case study, general practice, online, tool, data, evaluation, intervention, clinical

## Abstract

**Background:**

Research suggests that the rapid increase in worldwide antidepressant use is mainly due to a rise in long-term and potentially inappropriate use. It has been suggested that 1 in 3 antidepressant users among general practice patients are no longer experiencing clinical benefits from their medication and should commence deprescribing. However there are many barriers to antidepressant deprescribing for both patients and clinicians, which adds to the complex nature of reducing or ceasing the medication. As such, antidepressant deprescribing does not routinely occur in clinical practice. Evidence-based supports and interventions for safe and successful antidepressant deprescribing are needed to assist patients and their doctors. Interventions should also include an understanding of how an intervention works, why it works, and whom it is for.

**Objective:**

This study aims to evaluate how the WiserAD approach to antidepressant deprescribing works, whom it is for, and the underlying circumstances by (1) examining the experiences and perceptions of WiserAD among antidepressant users, (2) identifying the underlying mechanisms of the WiserAD approach to antidepressant deprescribing, and (3) describing in what contexts and to what extent the underlying mechanisms of WiserAD are suited for antidepressant users.

**Methods:**

A mixed methods case study with realist evaluation will be conducted among participants in the WiserAD randomized controlled trial for antidepressant deprescribing. Quantitative data will be obtained from up to 12 participants from the intervention and control arms at baseline and 3-month follow-up. Baseline data will be used to characterize the sample using descriptive statistics. Paired samples *t* tests will also be performed to compare responses between baseline and 3-month follow-up for participant self-management, skills, confidence and knowledge, beliefs about medicines, current emotional health, and well-being symptoms. Qualitative data from the same participants will be collected via narrative interview at 3-month follow-up. Quantitative and qualitative data will be converged to form a “case,” and analysis will be conducted within each case with comparisons made across multiple cases.

**Results:**

Recruitment of participants commenced in October 2022 and will be completed by March 2023. Analysis will be completed by June 2023.

**Conclusions:**

To our knowledge, this will be the first realist evaluation of an antidepressant deprescribing intervention in general practice. Findings from this evaluation may assist in the implementation of the WiserAD approach to antidepressant deprescribing in routine clinical practice.

**International Registered Report Identifier (IRRID):**

PRR1-10.2196/42526

## Introduction

### Background

Antidepressant use is rapidly increasing with the rate of antidepressant prescriptions doubling in western countries, such as Australia, Canada, the United Kingdom, and Iceland, over the past 10 years [1,2]. Antidepressants are the first-line treatment for depression that is considered as “more severe” (or moderate to severe depression), for which they have been shown to be effective [3-5]. Current guidelines advise that antidepressant treatment should continue for 6-12 months after remission of symptoms; however, research indicates that there has been an increase in long-term use (≥12 months) [6]. For example, in the Netherlands, long-term use increased from 30% in the period of 1995-2005 to 44% in the period between 2005 and 2015 [7] and from 45.6% to 67.4% between 2009 and 2010 in the United States [8]. Other studies have shown that the prevalence of long-term use among antidepressant users is approximately 36% and 42% in the United Kingdom [9] and the Netherlands [10], respectively.

Antidepressants are associated with common side effects such as gastrointestinal upset (for example nausea and constipation), dry mouth, and fatigue [11,12]. Research suggests that these initial side effects persist with long-term use [13], which also increases the risk of gastrointestinal bleeding [14], cardiovascular disease [15], weight gain [16], and feelings of emotional numbness [17]. Studies have shown that 1 in 3 people may be taking antidepressants without any clinical benefit [18,19], which suggests that prolonged use may place some people at unnecessary risk of adverse side effects.

The majority (86%) of antidepressants are prescribed in primary care [20], placing general practitioners (GPs) in a unique position to also deprescribe (the planned and supervised process of dose reduction or cessation [21]). However, antidepressant deprescribing can be complex and does not routinely occur in clinical practice [22,23] with reported barriers by both GPs and patients, including fear of relapse or recurrence and a lack of quality guidelines for deprescribing [24-27]. Discontinuation symptoms such as tremors, sweating, anxiety, mood swings, and electric shock sensations are also associated with stopping antidepressants [28-30] and can be confused with relapse or recurrence. As such, there is a need to support GPs and patients through the complexities of antidepressant deprescribing.

Patients have become increasingly responsible for managing the demands of their own health care [31,32] but are rarely being given the right support or information for how to do so effectively and confidently [31]. For deprescribing, GPs report only providing advice and support upon patient request [24,33]; hence, initiation of the deprescribing process is often left to the patient. However, patients who have approached their GP for antidepressant deprescribing often report becoming disillusioned with their clinician owing to a perceived lack of clinical skills and knowledge, causing them to turn to informal sources for deprescribing advice [34]. Stopping antidepressant medication without proficient GP support can increase the risk of withdrawal effects, relapse, and recommencement of medication [35,36]. As such, there is a need to determine how to best assist patients to make supported and evidenced-based decisions when stopping their antidepressant treatment in conjunction with their GP.

A web-based support tool called “WiserAD” has been developed to support patients and their GPs to safely and successfully deprescribe unnecessary antidepressant medication. WiserAD is currently being tested in a randomized controlled trial (RCT; see Textbox 1) and offers an opportunity to investigate the mechanisms and contextual factors that may influence the utility of the antidepressant deprescribing activities embedded in WiserAD. Determining how and why WiserAD works and for whom may assist in the implementation and sustainability of antidepressant deprescribing in clinical practice.

**Details about the WiserAD trial.**

WiserAD (Kaylor-Hughes et al, unpublished data, 2022) is a patient-centered, web-based structured support tool for patients and general practitioners (GPs) to safely deprescribe antidepressants while maintaining patients’ mental and physical well-being. WiserAD is based on the “5As” approach (ask, assess, advise, assist and arrange follow-up) to quitting smoking endorsed by the World Health Organization and the Royal Australian College of General Practitioners. Potential participants will be invited to consider taking part in the WiserAD randomized controlled trial by their GP clinic and will receive a follow-up call by a WiserAD team member who will provide more information about the study and check participant eligibility. Patients will be aged 18-75 years, stable on their selective serotonin reuptake inhibitor (SSRI) or serotonin–norepinephrine reuptake inhibitor (SNRI) for at least 12 months, have no or mild depressive symptoms, and have sufficient English to provide informed consent. Antidepressant users who are currently experiencing or expect to be experiencing a major life event in the next 3 months, are taking an SSRI or SNRI for a reason other than depression, are currently taking a non-SSRI or antipsychotic or another mood stabilizer, or do not have daily access to the internet will be excluded from participation. Once enrolled, participants will be randomly allocated to the WiserAD intervention or attention control group. Participants allocated to receive access to WiserAD will receive a login to the WiserAD portal, which will house their personalized tapering schedule and action plan for the management of any withdrawal symptoms, a daily mood tracker to monitor for changes in mental well-being and education about their antidepressant medication. Participants will only begin deprescribing once the tapering plan has been approved and discussed with their GP. Attention control participants will also be given a login to the WiserAD portal where they will only be able to view an antidepressant medication fact sheet. The WiserAD trial aims to recruit 312 antidepressant users from up to 30 GP clinics in Victoria, Australia.

### Objectives

The aim of this study is to understand how the WiserAD approach to antidepressant deprescribing works, for whom it is, and in what circumstances can it be implemented. To realize this aim, the following research questions will be answered: What are the experiences and perceptions of WiserAD by antidepressant users? What are the key underlying mechanisms of the WiserAD approach to antidepressant deprescribing? In what contexts and to what extent do the underlying mechanisms work for antidepressant users enrolled in WiserAD?

## Methods

### Theoretical Approach

A pragmatic, mixed methods case study with a core convergent design that draws upon the realist evaluation principles of Pawson and Tilley [37] will be used. Realist evaluation and case study designs are complimentary as both approaches aim to investigate how and why complex interventions work, not whether they work [38,39]. Mixed methods case studies can be used to examine a phenomenon from multiple perspectives in a real-life context [39], which allows for more in-depth understanding of a research problem [40]. For example, the exploration of qualitative and quantitative responses across patients who may have different levels of exposure to WiserAD will help to determine how the intervention may be working differently in different contexts and for different people [37]. In convergent mixed method designs, qualitative and quantitative data are collected concurrently and then merged together to enable comparison across and within multiple cases [40].

Realist evaluations are theory-driven evaluations that are based on an underlying theory of how an intervention (or program) works to trigger an outcome. In a realist evaluation, an initial theory is firstly elicited, and then it is tested and refined [37]. During the elicitation phase, a set of hypotheses (or initial program theories) are articulated using the formula “C + M = O,” where C refers to context, M to mechanism, and O to outcomes [37,38]. Mechanisms are underlying interactions between the resources of a program and the ways in which a participant interprets and responds to them. Mechanisms are central to realist evaluation as they provide an explanation for how and why programs produce outcomes [37,38,41]. Contexts are factors in situations that are not part of a program but interact, modify, and influence the program and how mechanisms may operate [37,38,41]. Outcomes are the intended and unintended consequences of a program and are generated by the activation of mechanisms and contexts [37].

**Figure 1:**
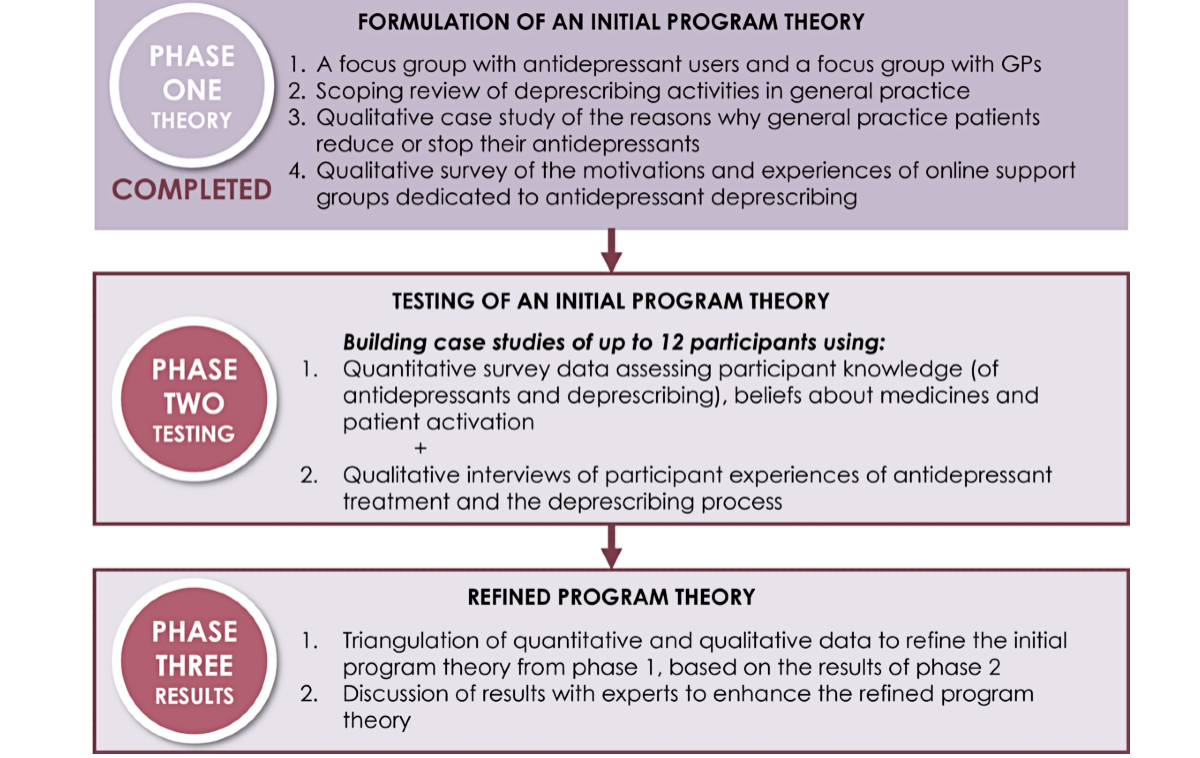
The 3 phases of a mixed methods case study with realist evaluation of the WiserAD approach to antidepressant deprescribing. GP: general practitioner.

Realist evaluations are conducted across three phases: (1) eliciting and formulating the initial program theory, (2) testing the initial program theory, and (3) building a refined program theory [37,42]. An initial program theory has already been formulated and is ready for testing in the current study. As such, phase 1 has been completed, and the focus of this protocol is how we propose to test the initial theory in phase 2 and present the results in phase 3 (see Figure 1). The RAMESES (Realist And Meta-narrative Evidence Syntheses–Evolving Standards) II reporting standards for realist evaluations [41,43] also informed the design of the 3 phases.

### Phase 1: Eliciting and Formulating the Initial Program Theory

Phase 1 has been completed. Briefly, 4 data sources were used to develop the initial program theory. First, separate focus groups with GPs (n=8) and individuals with a history of long-term AD use (current or past; n=9) were conducted in 2019 at the Department of General Practice, University of Melbourne (Kaylor-Hughes et al, unpublished data, 2022). The focus groups were analyzed thematically to identify barriers and facilitators to antidepressant deprescribing and inform the development of the first prototype of the WiserAD intervention. Second, a scoping review of 50 deprescribing interventions being used in general practice for any condition and medication was conducted in 2021 [44]. The scoping review identified key deprescribing activities and provided additional steps to create a self-sustaining deprescribing process loop for use in clinical practice. Third, a qualitative case study examined the reasons that 178 general practice patients with depressive symptoms gave for reducing or stopping their antidepressant medications (Coe et al, unpublished data, 2022). Thematic analysis was used to identify if the reasons why antidepressant users reduce or stop using antidepressants in a naturalistic setting could inform features of an antidepressant deprescribing intervention. Finally, a 2021 web-based qualitative survey was completed by 30 members of 2 web-based support groups for antidepressant deprescribing (Abid et al, unpublished data, 2022). This survey examined the motivations of participants for joining a web-based support group as well as their past and current experiences with deprescribing.

### Initial Program Theory

**Figure 2:**
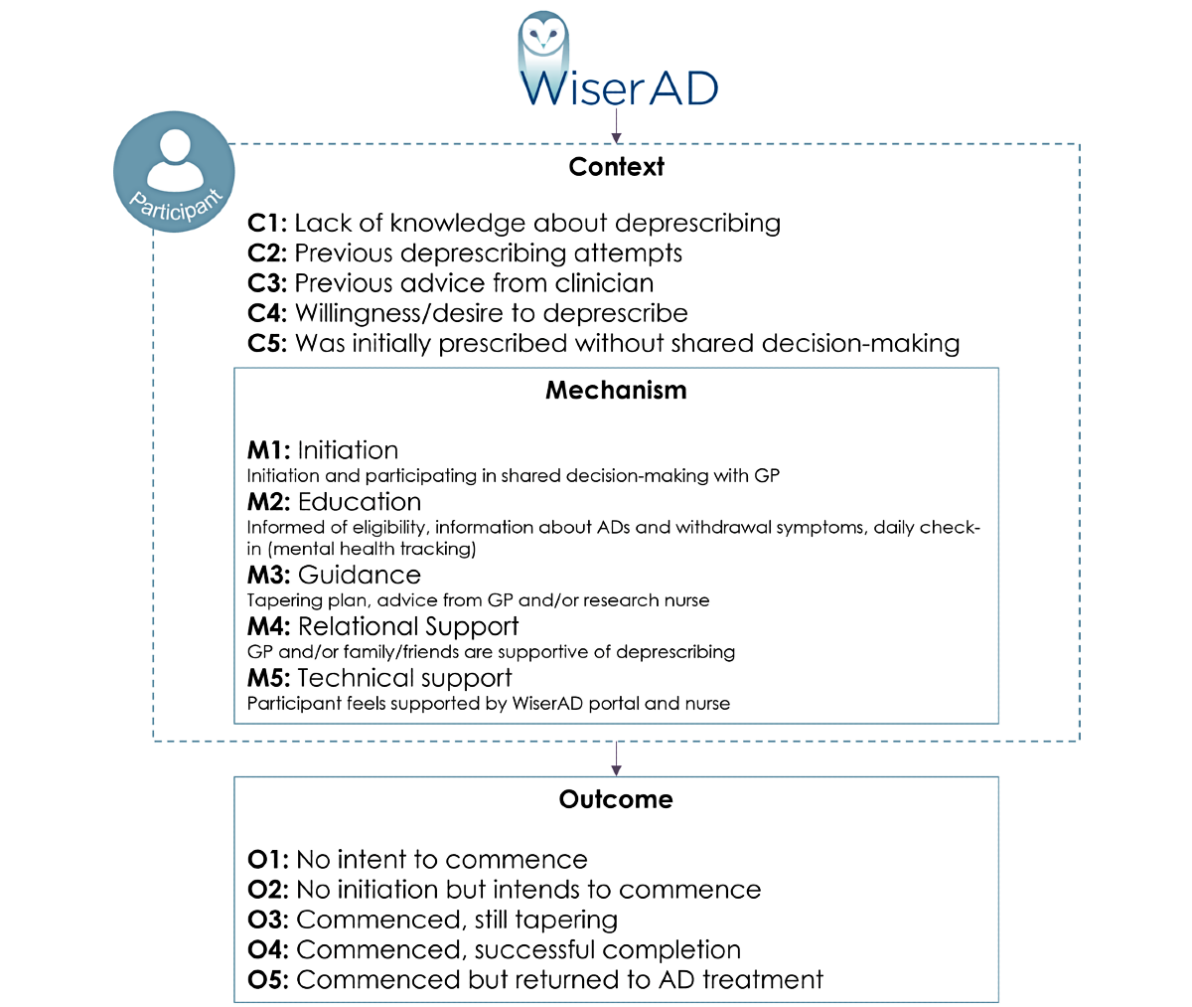
Conceptual context–mechanism–outcome framework for antidepressant deprescribing. AD: antidepressant; GP: general practitioner.

The findings from the studies in phase 1 have informed the initial program theory for how WiserAD may work, which is presented in the subsequent section (see Figure 2). It is expected that antidepressant users will have minimal prior knowledge of antidepressant deprescribing [26] (Coe et al, unpublished data, 2022). Participants may have also had limited clinical advice when initially being prescribed antidepressant medications with subsequent unsuccessful deprescribing attempts in the past [26] (Abid et al, unpublished data, 2022; Kaylor-Hughes et al, unpublished data, 2022). Despite the anticipated lack of deprescribing knowledge, it is anticipated that participants will be interested in or express a desire and willingness to deprescribe when presented with the opportunity [45,46].

The following potential WiserAD mechanisms are expected to trigger deprescribing: (1) initiation of and participating in shared decision-making with GP regarding antidepressant deprescribing [26,35,44,47]; (2) patient education (participants will be assessed for, and informed of, their eligibility to deprescribe on the basis of the stability of their mental well-being, information about antidepressant medication, and withdrawal symptoms and daily check-ins of their depressive symptom status) [26,44,48] (Abid et al, unpublished data, 2022; Kaylor-Hughes et al, unpublished data, 2022); (3) guidance (provided with a tapering plan that is supported by a GP and a research nurse) [26,35,44,47], (Kaylor-Hughes et al, unpublished data, 2022); (4) relational support (GPs, family, and friends are supportive of deprescribing) [26]; and (5) technical support (patient feels supported by the WiserAD tool and processes; for example, the research nurse) [26,44]. These mechanisms will work by increasing participant empowerment, confidence, and self-management and by positively challenging participant beliefs about antidepressant medication, allowing participants to at least intend to or have commenced deprescribing or successfully complete deprescribing.

### Phase 2: Testing the Initial Program Theory

Testing of the initial program theory will be conducted as a mixed methods case study realist evaluation. The realist evaluation will be carried out in the early stages (participant recruitment to 3-month follow-up) of the WiserAD trial and will form a multiple case study of up to 12 WiserAD participants from the intervention and control arms. Three-month follow-up has been chosen, as this will capture the context–mechanism–outcome configuration related to early decision-making by participants regarding the initiation of antidepressant deprescribing and the resulting outcome. The 5 outcomes presented in Figure 2 anticipate the different stages that participants may be in at 3-month follow-up. These outcomes acknowledge that participants may take longer or shorter periods of time to taper their medication. As this evaluation focuses on how, why, and who WiserAD works for, it will determine all possible outcomes of an approach to antidepressant deprescribing rather than showing if it works. The effectiveness of WiserAD on successful deprescribing will be shown at the completion of the WiserAD RCT where an additional evaluation may be carried out and presented in a future publication.

### Recruitment and Consent

All participants will have been invited to complete an interview at the time of enrollment in the trial. Participants from the intervention and control arms who agreed to an interview will then be purposively selected on the basis of their age, gender, and level of use of the web-based WiserAD tool (ie, the number of logins to the website in the 3 months since enrollment into the trial) and their GP clinic to ensure diversity of experiences. When participants reach 3 months post commencing participation in the trial, author AC will send an email with a plain-language statement to reinvite them to an interview. AC will then follow up the email with a phone call within 7-10 days. Interested participants will then be booked in to complete the interview at a mutually convenient time.

### Data Collection

Quantitative data from the WiserAD study collected at baseline and 3-month follow-up from interview participants will be used in this study. Surveys will be completed digitally, though, if required, the surveys can be completed via telephone or video call or in person at the participants GP clinic. The baseline survey will collect demographic information and both the baseline and 3-month follow-up surveys will ask questions about participant self-management, skills, confidence, and knowledge (Patient Activation Measure–Mental Health) [49], beliefs about their antidepressants (Beliefs About Medicines Questionnaire) [50], and current emotional health and well-being symptoms (Patient Health Questionnaire-9) [51], including those of generalized anxiety disorder; (7-item Generalized Anxiety Disorder scale [52]). WiserAD website usage data will be analyzed to determine the number of logins, number of times pages were looked at, and how much time was spent on each page. The survey has been tested and approved by antidepressant users with lived experience of depression.

Interviews with WiserAD participants will be conducted by a PhD researcher (AC) at 3-month follow-up to further identify and understand the mechanisms of impact that WiserAD has on antidepressant deprescribing. Interviews will be conducted via telephone, video call, or in person. Each interview will last approximately 60 minutes. A narrative interview approach will be taken to allow the participant to naturally report potential mechanisms, contexts, and outcomes without the risk of interviewer bias. The interview process will be guided by the narrative interviewing phases, as suggested by Jovchelovitch and Bauer [53], namely, preparation (formulation of questions), initiation (posing or formulating the topic for narration), main narration (allowing interviewee to talk without interruption), questioning phase (prompting interviewee to continue narration), and conclusion of talk [53]. The phases of narrative interviewing are designed to elicit rich narration rather than falling into a pattern of question-answer with the interviewee.

### Sample Size

When conducting qualitative studies, a sample size is deemed adequate when no new information (or data saturation) has been reached [54]. However, for realist evaluations, reaching saturation occurs by exploring a combination of qualitative and quantitative data along with the information obtained when formulating the initial program theory [55]. Additionally, the descriptive statistics that will be generated in this study do not require a minimum sample size. This study will be guided by the case study design recommendation of a minimum sample size of 4-10 participants [40]. As this is a novel area of research, a target sample size of 10-12 participants will used to ensure thoroughness and depth.

### Data Analysis

#### Quantitative Data

Quantitative data (numerical and closed-question data) will be coded and prepared for analysis in Stata (version 17; StataCorp) [56]. Summary statistics in the form of descriptives (means and SDs for continuous data and frequencies and percentages for categorical data) will be calculated, and repeated measures *t* tests will be performed to compare survey data at baseline and 3-month follow-up. Data will be described and graphically represented. Identification of missing values will first be achieved through web-based assessment, and the Little Missing Completely at Random test [57] will be performed.

#### Qualitative Interview Data

Narrative interviews will be audio-recorded and transcribed verbatim. Anonymized transcripts will be uploaded to NVivo (version 12; QSR International) [58] for management, coding, and analysis. Data will be coded as a context, mechanism, or outcome. Coding, analysis, and interpretations will be conducted iteratively via discussion among study team members. Independent double-coding of the transcripts will be completed by 2 study team members.

#### Data Converging

**Figure 3:**
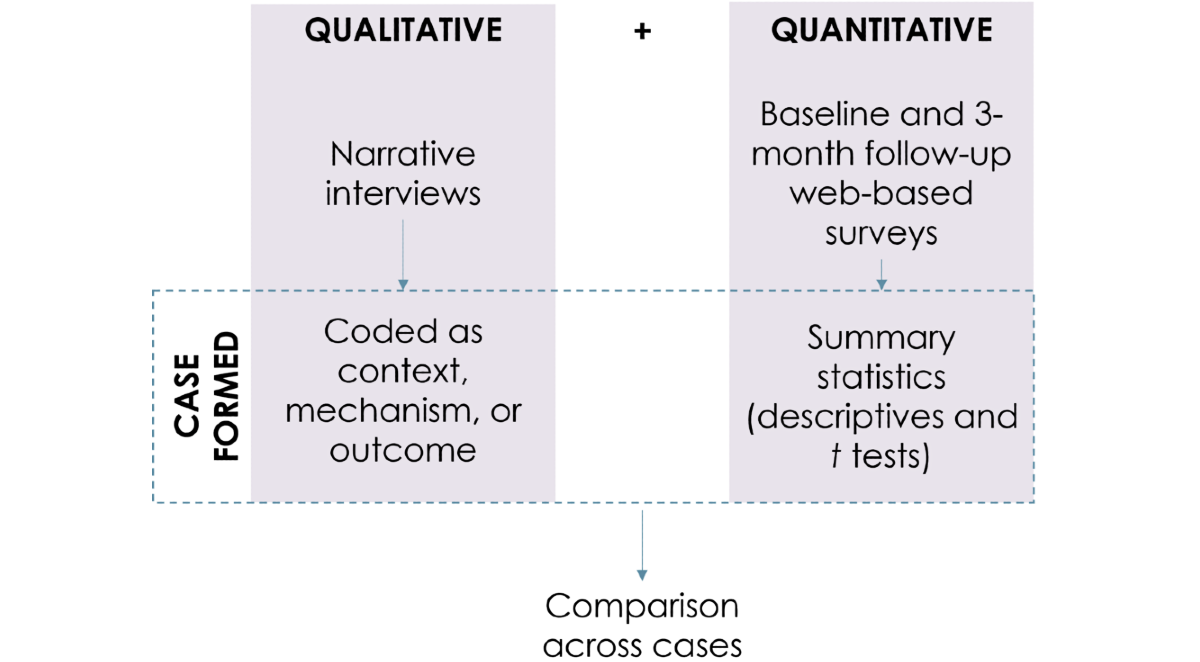
Concurrent triangulation of data.

In accordance with convergent mixed method designs, qualitative and quantitative data will be collected and analyzed concurrently to generate cases (the main subject of study in a case study) [39,40]. These cases will represent an individual participant in the WiserAD trial. Cases will be analyzed separately and then compared across cases to determine any similar or opposing evidence through data triangulation and theme matching (Figure 3).

### Phase 3: Building a Refined Program Theory

In phase 3, triangulation of the quantitative and qualitative data from phase 2 will be used to check and, if necessary, adapt the initial program theory to create a refined program theory showing what works, for whom, and in what circumstances, for successful antidepressant deprescribing. The refined theory will be discussed in depth by the WiserAD research and investigator team that comprises experts in primary care, nursing, health economics, psychiatry, psychology, pharmacology, and business management. Experts will provide validation or disconfirmation of the program theory on the basis of their own experiences and knowledge. This feedback will be used to apply final refinements to the theory. The final refined theory will inform any necessary changes to the web-based WiserAD support tool prior to implementation into clinical practice.

### Ethical Considerations

Ethical approval for this study has been granted by the University of Melbourne Human Ethics Committee (#20558). Participants will receive a plain-language statement that will detail the aims of the study, what participation involves and, information regarding privacy and confidentiality of data. Confirmation of consent will be given verbally at the time of the interview, which will be audio-recorded with the participants’ permission. Consent to take part in the interview will also be indicated by continued participation in the interview. Participants will be asked for permission to have their interview audio-recorded and will be informed that they are free to decline, and if they consent, they are free to discontinue the recording or interview at any time. No participant details will be stored with the audio recordings or transcripts, both of which will receive a study identification number. Participants will be informed that any names mentioned in the interviews will be anonymized in the transcript. All quantitative data will be deidentified prior to being provided to AC for analysis by the WiserAD data manager. After completing the interview, participants will receive an Aus $50 (US $33.5) gift card as reimbursement for their time.

## Results

The WiserAD trial commenced in May 2022. Sample size requirements for the realist evaluation were reached by November 2022 with the anticipated completion date for the current study being March 2023. Dissemination of the study findings will occur via peer-reviewed publications, public presentations, and a PhD thesis in 2023.

## Discussion

### Expected Findings

This protocol presents a mixed methods case study with realist evaluation of the web-based WiserAD support tool. This will be an evaluation of the first participants in the WiserAD RCT and aims to understand how the WiserAD approach to antidepressant deprescribing works, for whom it is intended, and in what circumstances can it be implemented. Quantitative survey data and qualitative interview data will provide information about participants’ experiences and perceptions of WiserAD to confirm and refine an initial theory of how antidepressant deprescribing may work in general practice. It is anticipated that initiation of deprescribing, guidance, provision of education about deprescribing, relational support, and technical support will be drivers for patients to intend to or be actively deprescribing their antidepressant.

Only one realist evaluation of a deprescribing intervention has been conducted to date, which investigated the impact of providing an educational brochure about benzodiazepines to older adults in the community [46]. Martin and Tannenbaum [46] reported that by improving knowledge about medication, patients’ self-efficacy to deprescribe also increased. As detailed earlier in this protocol, it is expected that education and increased self-efficacy will also impact patients’ decision to deprescribe their antidepressants. To our knowledge, this will be the first realist evaluation of an antidepressant deprescribing intervention in general practice and will contribute empirical and theory-informed novel findings about the mechanisms underlying antidepressant deprescribing. It will advance knowledge of antidepressant user experiences of deprescribing in general practice and provide a theoretical contribution to the deprescribing literature. Specifically, it will help determine what general practice patients need in order to successfully and safely deprescribe their antidepressant. It will also enhance knowledge about how to support patients to make decisions about their own antidepressant treatment.

### Strengths and Limitations

This study sample will satisfy the sample size requirements of a case study design; however, a sample of 12 participants is small. Additionally, the duration of antidepressant deprescribing may occur over a time period that is longer than 3 months. Therefore, future evaluation of the WiserAD approach to deprescribing should be conducted upon completion of the RCT. The use of mixed methods over a singular data collection method is a strength of this study and will allow for a better understanding of participants’ experiences with the WiserAD approach to antidepressant deprescribing. Mixed methods designs are also used to provide in-depth, rigorous evidence of a phenomenon; thus, we can be confident that the findings of this study will make an important contribution to the literature.

### Conclusions

This will be the first realist evaluation of an approach to antidepressant deprescribing in general practice. The findings from this study will provide insight into patients’ experiences and perceptions of antidepressant deprescribing and thus increase the current understanding of the factors that influence the occurrence of deprescribing in clinical practice.

## Abbreviations

GP: general practitioner
RAMESES: Realist And Meta-narrative Evidence Syntheses–Evolving Standards
RCT: randomized controlled trial

## Data Availability

The data sets generated or analyzed in this study will not be publicly available as the proposed sample size is small and could compromise the privacy of research participants. Additionally, consent and ethical approval for this study does not include a provision for the sharing of data from this study.
